# Regional BCG vaccination policy in former East- and West Germany may impact on both severity of SARS-CoV-2 and incidence of childhood leukemia

**DOI:** 10.1038/s41375-020-0871-4

**Published:** 2020-06-18

**Authors:** Julia Hauer, Ute Fischer, Franziska Auer, Arndt Borkhardt

**Affiliations:** 1 Department of Pediatrics, Pediatric Hematology and Oncology, University Hospital Carl Gustav Carus, Dresden, Germany; 2 National Center for Tumor Diseases (NCT), Partner Site Dresden, Dresden, Germany; 3 Department of Pediatric Oncology, Hematology and Clinical Immunology, Medical Faculty, Heinrich-Heine University, Düsseldorf, Germany; 4 German Cancer Research Center (DKFZ), Heidelberg, Germany

**Keywords:** Cancer epidemiology, Infectious diseases

## To the Editor:

We read with great interest the recent thorough study from Miller et al. [1], who reported a beneficial impact of early Bacillus Calmette–Guérin (BCG) vaccination, and linked morbidity and mortality due to SARS-CoV-2 with BCG vaccination policy. They found that countries without policies of universal BCG vaccination (like Italy, the Netherlands, or the United States) have been more severely affected compared with countries with universal and long-standing BCG policies. Since senior citizens are particularly sensitive to COVID-19, newborn vaccination programs implemented in the 1940s and 1950s should be the most beneficial. Thus, we were impressed by the linear correlation between the year of the establishment of universal BCG vaccination and the mortality rate presented by Miller et al. [1]. In our view, this is a convincing argument for the hypothesis that the earlier the vaccination policy was established, the larger the segment of the elderly population being protected.

**Fig. 1:**
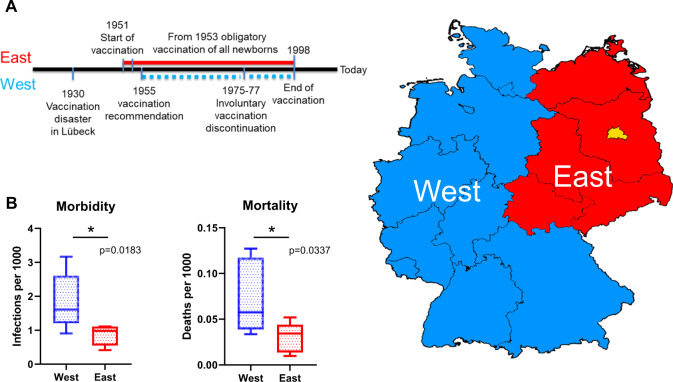
Morbidity and mortality due to SARS-CoV-2. **a** Vaccination policy in East and West Germany. When BCG vaccination was first introduced against tuberculosis in Germany (in the city of Lübeck) in 1930, tragically 131 newborns fell ill, and 77 died due to a contamination of the vaccine with virulent tubercle bacilli. This was the greatest vaccination disaster of the twentieth century, and delayed introduction of BCG vaccination in Germany till after World War II. **b** Morbidity and mortality due to SARS-CoV-2 in federal states formerly belonging to East and West Germany, excluding Berlin. Data were extracted from Johns Hopkins University (as per 28.4.2020). Population numbers were extracted for the federal states as per December 2018 (www.statista.com). *p*-values were calculated using the Studentʼs *t*-test.

We would like to make the scientific community aware of a unique historical circumstance in Germany, where divergent BCG vaccination policies existed in the politically divided country (1949–1989) before German reunification in 1990. In East Germany, BCG vaccination programs were established by the communist government in 1951, and soon became compulsory in 1953, leading to near-universal (99.8%) BCG vaccination of newborns by day 3. By contrast, voluntary BCG vaccination (recommended since 1955) was far less common in West Germany, due to low incidence of the disease after the Second World War. In early years, only 7–20% of all newborns became BCG-vaccinated in Western Germany, with almost complete cessation of vaccination between 1975 and 1977 (Fig. 1a). Thus, we believe that the comparison of morbidity and mortality of SARS-CoV-2 would be particular informative in the light of the rather uniform genetic, social, and cultural background. Here, we record those data in formerly East and West German federal states (excluding Berlin, Fig. 1b). Our observations strongly support the analysis from Miller et al., and point toward BCG vaccination having a protective effect. We did not observe a significant difference in the lethality of SARS-CoV-2; once people became infected, the course of disease is not significantly different between formerly East and West German parts of the reunified country. It is fair to conclude that, 30 years after German reunification, the standard of intensive care has been completely harmonized throughout the country, and at the time of our analysis, the health care system in Germany still had large capacities. Besides BCG vaccination, we cannot exclude other factors influencing morbidity and mortality, e.g., the speed of the pandemic spread, differences in the social behavior, or the potentially greater likelihood of being infected with SARS-CoV-2 in the larger cities of Western Germany. We also acknowledge the fact that statistical correlation does not prove causation, but SARS-CoV-2 testing capacities, a frequent confounding factor when comparing different populations across countries, were early on expanded to far more than 100,000/day (end of March 2020) all over Germany. Furthermore, demographic characteristics show that relatively more elderly people live in East Germany than in West Germany. The age distribution shows that the proportion of people above 64 years of age is generally higher in Eastern federal states formerly belonging to the German Democratic Republic (GDR) [2]. Thus, age-wise, the population with a greater risk to succumb due to COVID-19 is even more abundant in East, compared with West Germany.

We would like to remind readers that a correlation between BCG vaccination and reduced disease risk beyond tuberculosis has long been suspected. In particular, many older studies link BCG vaccination to a lower cancer risk, and there was even an international symposium held in 1982 entitled “BCG Vaccination against Cancer and Leukemia” (Chicago). Mortality related to childhood leukemia was reported to be significantly reduced in a retrospective study of 54 414 BCG-vaccinated versus 172 986 non-vaccinated infants in Chicago [3]. The question was brought up again in 1978 in a meta-analysis of Rosenthal’s data, and two other cohorts confirmed the initial observation [4]. With respect to childhood leukemia, the potentially beneficial effects of the East German BCG vaccination program were also reported by Spix et al. [5]. The authors compared the rate of childhood leukemia before and after German reunification. Somewhat counterintuitively, the rate of childhood leukemia was clearly lower in the former GDR (East Germany) with 3/100,000 children compared with 3.7/100,000 in former West Germany, but reached West German levels ~8 years after reunification with four cases of acute lymphoblastic leukemia (ALL)/100,000 children below 15 years of age. It should be emphasized that cancer registries were exemplary in the former GDR, and thus this “catch-up effect” cannot be attributed to poor registration. In addition, meta-analysis of several epidemiological studies has shown that protection from childhood ALL, the most common malignancy in children, is observed only when children are vaccinated with BCG very early in life (<3 months) [6].

In recent years, a new immunological concept—trained immunity—has emerged, improving our understanding of the role of BCG vaccination in shaping the innate immune memory response. Innate immune cells, such as macrophages, monocytes, or NK cells, can change their epigenome after exposure to infection, vaccination, or other stressors, which modifies their expression profile and cell physiology [7]. BCG vaccination in particular, educates hematopoietic stem cells and generates trained monocytes and macrophages [8]. In a double-blinded, placebo-controlled study with healthy male subjects who were challenged by attenuated Yellow Fever Virus after having received a BCG vaccine or placebo, the viremia was significantly lower in the vaccinated group [9]. Besides infection with SARS-CoV-2 and childhood ALL, there may be many other malignant and nonmalignant, e.g., infectious diseases for which measures that augment our innate immune responses are beneficial [10, 11]. However, it remains unclear to date whether BCG vaccination across different age groups, e.g., newborns, adolescents, young, and elderly people, has uniformly the same beneficial effect.

With regard to the current SARS-CoV-2 pandemic situation, we fully support the idea of implementing controlled clinical trials for BCG vaccination, as already started by Curtis et al. [12]. Only well-controlled clinical trials may prove a benefit of this old vaccine that was developed by the two French immunologists almost 100 years ago. Low- and medium-income regions like Africa or India may benefit mostly if such trials prove a clinical benefit, at least until a specific and effective SARS-CoV-2 vaccine becomes available.
